# Bimetallic Pt-Ni Nanoparticles Confined in Porous Titanium Oxide Cage for Hydrogen Generation from NaBH_4_ Hydrolysis

**DOI:** 10.3390/nano12152550

**Published:** 2022-07-25

**Authors:** Yuqian Yu, Li Kang, Lixian Sun, Fen Xu, Hongge Pan, Zhen Sang, Chenchen Zhang, Xinlei Jia, Qingli Sui, Yiting Bu, Dan Cai, Yongpeng Xia, Kexiang Zhang, Bin Li

**Affiliations:** 1 Guangxi Key Laboratory of Information Materials, Guangxi Collaborative Innovation Center for Structure and Properties for New Energy and Materials, School of Material Science and Engineering, Guilin University of Electronic Technology, Guilin 541004, China; yuyuqian997@163.com (Y.Y.); kangli000hello@163.com (L.K.); 15755028821@163.com (Z.S.); Zhang_linba_3760@163.com (C.Z.); jia15292079581@163.com (X.J.); sqlguet123@163.com (Q.S.); ytb1172701255@163.com (Y.B.); dancai1985@guet.edu.cn (D.C.); ypxia@guet.edu.cn (Y.X.); kxzhang@guet.edu.cn (K.Z.); li_bin@guet.edu.cn (B.L.); 2 School of Mechanical & Electrical Engineering, Guilin University of Electronic Technology, Guilin 541004, China; 3 School of New Energy Science and Technology, Xi’an Technological University, Xi’an 710021, China

**Keywords:** hydrogen generation, porous titanium oxide cage, PtNi nanoparticles, sodium borohydride hydrolysis

## Abstract

Sodium borohydride (NaBH_4_), with a high theoretical hydrogen content (10.8 wt%) and safe characteristics, has been widely employed to produce hydrogen based on hydrolysis reactions. In this work, a porous titanium oxide cage (PTOC) has been synthesized by a one-step hydrothermal method using NH_2_-MIL-125 as the template and L-alanine as the coordination agent. Due to the evenly distributed PtNi alloy particles with more catalytically active sites, and the synergistic effect between the PTOC and PtNi alloy particles, the PtNi/PTOC catalyst presents a high hydrogen generation rate (10,164.3 mL∙min^−1^∙g^−1^) and low activation energy (28.7 kJ∙mol^−1^). Furthermore, the robust porous structure of PTOC effectively suppresses the agglomeration issue; thus, the PtNi/PTOC catalyst retains 87.8% of the initial catalytic activity after eight cycles. These results indicate that the PtNi/PTOC catalyst has broad applications for the hydrolysis of borohydride.

## 1. Introduction

The overconsumption of traditional fossil fuels has brought in severe energy shortages and environmental pollution issues, such as the greenhouse effect [1]. To solve the above issues, hydrogen energy, as an efficient and sustainable energy, is considered to be a promising alternative to fossil energy [2,3]. The extensive development and use of hydrogen energy is conducive to the pursuit of carbon neutrality and emission peak. Hydrogen produced by the hydrolysis of sodium borohydride (NaBH_4_) has been regarded as one of the most promising hydrogen production methods due to the advantages of high theoretical hydrogen production density (10.8 wt%), low hydrogen release temperature, controllable reaction process, high hydrogen purity, and environmental friendliness [4]. However, the slow hydrolysis reaction limits the wide use of hydrogen.

In order to accelerate the reaction kinetics of hydrolysis NaBH_4_, a variety of catalysts such as Co [5,6,7,8], Ni [9,10,11], Rh [12], Pd [13], Ru [14,15], and Pt [16,17] have been comprehensively studied. Although Pt-based catalysts are one of the most active catalysts, the scarce storage and expensive price are the main obstacles to their large-scale application. Therefore, increasing the utilization efficiency of Pt remains the focus of the search. Previous studies have demonstrated that combining Pt with non-noble metals (such as Co [18,19], Ni [20,21,22], and Fe [23,24]) could significantly improve the utilization efficiency of the catalysts. For example, Shumin Han et al. synthesized a carbon nanosphere (CNS)-supported ultrafine bimetallic Pt-Co nanoparticle (CNSs@Pt_0.1_Co_0.9_) catalyst for NaBH_4_ catalysis. The as-prepared CNSs@Pt_0.1_Co_0.9_ catalyst exhibited excellent performance in kinetic and thermodynamic tests [25]. Younghun Kim et al. designed a magnetic core and multi-shelled silica/titania-supported bimetallic (Pt/Ni NPs Fe_3_O_4_@SiO_2_@TiO_2_) catalyst for catalyzing the hydrolysis of NaBH_4_ [20]. Jong-Sung Yu et al. uniformly deposited PtFe hydroxide by in situ hydrolysis of urea, followed by the preparation of a carbon-supported PtFe catalyst in ethylene glycol, and the catalyst exhibited excellent electrocatalytic performance [24].

Recently, Ni combined with precious metals, such as Pt-Ni and Ru-Ni, have been confirmed to be effective catalysts for hydrogen production from hydrolysis NaBH_4_ [26,27,28]. However, these catalysts exhibit poor catalytic activity due to the accumulation of metal nanoparticles (NPs) during the reaction. Strategies including structural and morphology control, as well as the addition of suitable carries can effectively inhibit the agglomeration problem [29,30,31]. In addition, the introduction of support material not only is conducive to the distribution of metal NPs but also improves the metal properties through geometric and electronic effects. Anelia Kakanakova-Georgieva et al., employing theoretical calculations, demonstrated that the porous structural material and the synergistic effect between metal NPs with support materials played an important role in the activity of the catalyst [32,33]. Porous hollow structures assembled from nanosheets with large surface areas could provide a unique microenvironment both on the inside and outside through species channels for guest shuttling [34]. Among numerous porous materials, metal-organic frameworks (MOFs) with tunable metal ions and organic ligands are extensively searched in the fields of energy storage and catalysis [35]. Furthermore, they also act as a self-sacrificing template in preparing the porous hollow materials. For example, pioneering studies used the Ti-MOFs as carriers to improve the catalytic performance of metal catalysts for hydrogen production [36,37]. Therefore, reasonably designed porous structural carries for the dispersion of metal NPs enables the achievement of a satisfactory catalytic performance.

Herein, PtNi NPs were confined in a porous titanium oxide cage (PTOC) derived from NH_2_-MIL-125 (Ti) by a facile hydrothermal method and used for the hydrogen production of hydrolysis NaBH_4_. The synthesized catalysts exhibit good catalytic activity with a high hydrogen generation rate (10,164.3 mL∙min^−1^∙g^−1^) and low activation energy (28.7 kJ∙mol^−1^). In addition, the robust porous structure of PTOC benefits from the distribution of PtNi alloy particles and suppresses the agglomeration issue; thus, the PtNi/PTOC nanocomposite catalyst retains 87.8% of the initial catalytic activity after eight cycles.

## 2. Materials and Methods

### 2.1. Materials

All chemicals were of analytical grade and used without further purification. 2-aminoterephthalic acid, sodium borohydride (NaBH_4_), and nickel nitrate hexahydrate (Ni(NO_3_)_2_·6H_2_O) with a purity of 99% were purchased from Alfa Aesar Co., Ltd. (Tianjin, China). Chloroplatinic acid hexahydrate (H_2_PtCl_6_·6H_2_O), titanium (IV) isopropoxide, L-alanine, and dimethylformamide (DMF)were purchased from Aladdin Reagent (Shanghai, China). All experiments were performed using DMF and anhydrous CH_3_OH as solvents.

### 2.2. Synthesis of NH_2_-MIL-125

The preparation of NH_2_-MIL-125 nanocrystals followed a previously reported process [38]. Using DMF and ethanol as organic reaction solvents, 2-aminoterephthalic acid (500 mg, 2.76 mmol) was dissolved in a mixture solvent (10 mL) of 1 mL of CH_3_OH (1 mL) and DMF (9 mL). Subsequently, 0.76 mmol of titanium isopropoxide was slowly added to the mixture under ultrasound. The solution was then placed in a 25 mL Teflon-lined reactor and heated at 150 °C for 72 h. After, the mixture was cooled to room temperature and the yellow powder was recovered by centrifugation. To remove impurities, the collected powder was washed sequentially with DMF, ethanol, and deionized water and dried at 80 °C for 12 h.

### 2.3. Synthesis of PTOC

NH_2_-MIL-125 (10 mg) was sonicated and dispersed in 5 mL of anhydrous ethanol. Next, 47.5 mg of L-alanine was added to the mixture and stirred for 6 h. The solution was then placed in a reaction vessel containing 25 mL of Teflon liner and heated at 176 °C for 36 h. The white precipitate was recovered by centrifugation, washed with ethanol, and dried under vacuum at 80 °C for 12 h.

### 2.4. Preparation of PtNi/PTOC

Amino acid molecules (generally a class of mild reducing agents) are used to prepare metal NPs. Herein, NH_2_-MIL-125 (10 mg) was sonicated and dispersed in 5 mL of anhydrous ethanol. Then, L-alanine (47.5 mg) was added into the mixture and stirred for 6 h. H_2_PtCl_6_·6H_2_O (1.0 mg, 2.3 µmol) and Ni(NO_3_)_2_·6H_2_O (3 mg, 10.3 µmol) were added sequentially and stirred for 1 h. Then, the mixture was placed in a 25 mL Teflon pan and heated at 176 °C for 36 h. After centrifugation, the mixture was dried with ethanol at 80 °C for 12 h. For the comparison, Ni(NO_3_)_2_·6H_2_O was not added in the preparation process of Pt/PTOC, and H_2_PtCl_6_·6H_2_O was not added to Ni/PTOC; the other steps were consistent with the preparation process of PtNi/PTOC.

### 2.5. Characterization

The morphology of the PtNi/PTOC catalyst was analyzed by scanning electron microscopy (SEM, Quanta 200, FEI, Hillsboro, OR, USA) under a vacuum environment and 30 kV AC voltage. The test sample was dispersed on conductive material and stuck on a small sample holder. Excess powder was blown off with gas to avoid contaminating the cavity. The morphology and elemental composition of the catalyst was analyzed using a transmission electron microscope (TEM, JEOL 2010, JEOL, Tokyo, Japan) and dispersive X-ray detector (EDX) with an informal resolution of 0.12 nm and a point resolution of 0.25 nm. The powder was put into an anhydrous ethanol solution, shaken well with ultrasonic waves, and dropped onto the microgrid support film to obtain the sample to be tested. The chemical structure of the catalyst was characterized by Fourier-transform infrared (FT-IR) spectroscopy (Nicolet 6700, Waltham, MA, USA) in the wavenumber range of 400–4000 cm^−1^. The fine powder of the sample was uniformly dispersed in potassium bromide in the ratio of 1:100 (m_catalyst_:m_KBr_) and the transparent flakes were obtained by the tablet method at the pressure of 5 MPa for 30 s. The crystal structure was analyzed by X-ray diffraction (XRD, 1820, Philips, Amsterdam, The Netherlands), with a scan angle from 5° to 90°, a step size set to 0.02, a working voltage of 40 kV, and a working current of 40 mA. The sample preparation was carried out as follows: the powder sample was evenly distributed in the sample holder and compacted with the glass plate. The sample surface was required to be smooth and flush with the glass surface. The nitrogen-desorption isotherms of the PtNi/PTOC catalysts were investigated using a QuantachromeAutosorb-iQ2 adsorber. The specific surface area of PtNi/PTOC catalyst was determined using a fully automated ratio meter and porosity analyzer. The samples were degassed in a glass tube at 150 °C for 10 h and then analyzed in liquid nitrogen. The pore size of a pore of the PtNi/PTOC catalyst was determined by the BJH method. X-ray photoelectron spectroscopy (XPS; Thermo Electron ESCALAB 250, Waltham, MA, USA), was mainly used qualitatively and semi-quantitatively through the analysis of catalysts, the valence state, species class, and surface content. The sample was pressed on aluminum foil, the excitation light source was Al Kα (hv= 1486.6 eV), and the final XPS was calibrated by C 1s (284.8 eV).

### 2.6. Hydrogen Production Testing

The catalytic hydrogen generation experiments were measured on a self-built hydrogen generation device [39]. The volume of hydrogen produced was determined by the equivalent displacement of water. First, 0.1 g of catalyst was added in a 125 mL conical flask. Next, 10 mL of the solution containing 1.5 wt% NaBH_4_ and 5 wt% NaOH was injected into the conical flask. The produced gas was collected in a container filled with water after flowing through a condenser and dryer to remove water vapor. The volume of produced H_2_ was measured by the water displacement method. The water was displaced into a 1 L flask through a tube connected with a gas-gathering container and weighted by an electronic balance (UX2200H, Shimadzu Corporation, Kyoto, Japan). A computer connected to the electronic balance was used to record water quality automatically. The hydrogen released per gram of catalyst per unit time (HGR) was calculated through the display on the computer. After one hydrolysis test was completed, the catalyst was immediately washed and dried for 12 h. Subsequently, a fresh 10 mL of the 1.5 wt% NaBH_4_ and 5 wt% NaOH solution was added to repeat the above measurements.

The hydrogen generation rate (HGR) was calculated according to the following equation:

$$HGR=\frac{V_{H_{2}0}\left(\mathrm{mL}\right)}{t(\min)\times m\left(g\right)}$$

where $V_{H_{2}0}$ is the volume of drained water, *m* is the total mass of the catalyst, and *t* is the total reaction time in minutes [40].

## 3. Results and Discussion

### 3.1. Catalyst Characterization

**Figure 1:**
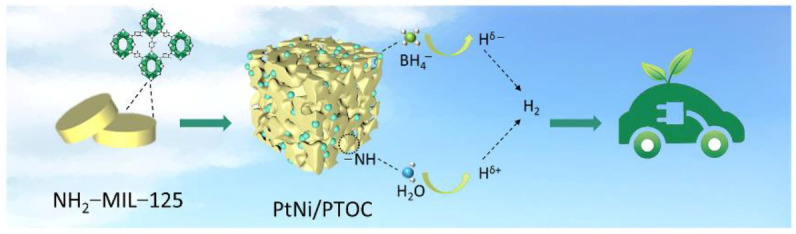
The illustration of the synthetic route of PtNi/PTOC.

In this paper, PtNi/PTOC was synthesized by a simple hydrothermal method and wet-reduction method. Figure 1 shows a schematic diagram of the preparation of PtNi/PTOC (PTOC). First, a round cake of NH_2_-MIL-125 (Ti) was obtained using 2-aminoterephthalic acid as organic ligands and titanium (IV) isopropoxide as a metal precursor. Then, PTOC with a porous hollow structure was formed into an alcoholic thermal process at 176 °C under auxiliary amino acid molecules L-alanine. Lastly, PtNi precursors were reduced to Pt_3_Ni NPs by the L-alanine. The formation of PTOC involved the Kirkendall effect of Ti ion dissolution and recrystallization. The Ti(iv) ions were firstly dissolved from NH_2_-MIL-125(Ti) nanocrystals by coordination of Ti(iv) with amino acids (l-alanine), leading to the formation of sheet-like titanium oxide NPs on the NH_2_-MIL-125(Ti) nanocrystals. With this continuous transformation, successive shells of titanium oxides were generated and transferred into the completed porous cages.

**Figure 2:**
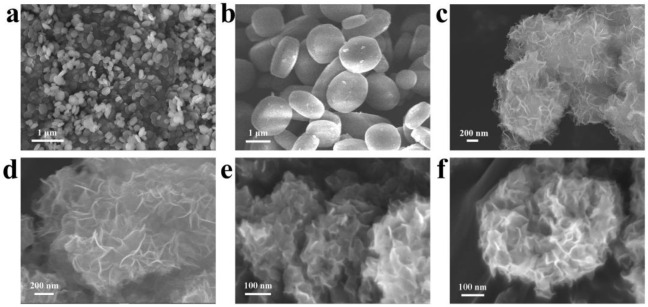
SEM images of (**a**,**b**) NH_2_-MIL-125; (**c**,**d**) PTOC and (**e**,**f**) PtNi/PTOC catalyst.

The morphology of NH_2_-MIL-125 and PTOC were characterized by SEM; as can be seen in Figure 2a,b, the NH_2_-MIL-125 exhibits a round cake with a smooth surface, and the size is around 300–500 nm. After the auxiliary of the amino acid molecule L-alanine under hydrothermal circumstances, the cage structure of PTOC remains, with multi-channel interlacing on the surface (Figure 2c,d). Due to the alcoholization of NH_2_-MIL-125, the nanosheets were assembled into a cage structure. L-alanine is commonly used as a mild reducing agent for the preparation of metal NPs [41,42]. After the hydrothermal reaction, PtNi precursors were reduced to Pt_3_Ni NPs and confined in PTOC. As shown in Figure 2e,f, compared with the PTOC sample, Pt-Ni/PTOC still retained the unique “nanocage” structure.

**Figure 3:**
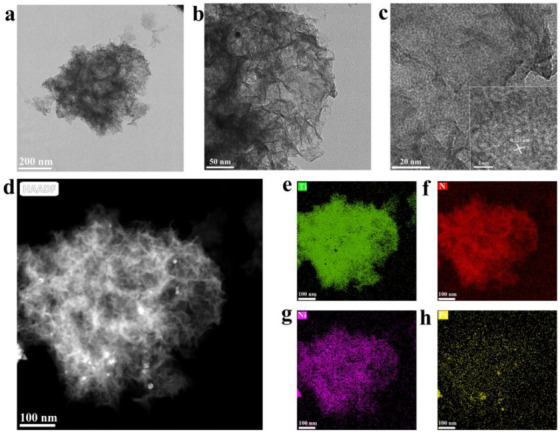
TEM images of (**a**,**b**) PtNi/PTOC catalyst; (**c**) HRTEM; (**d**) HADDF-STEM (**e**–**h**); EDX images of PtNi/PTOC.

As shown in Figure 3, HRTEM analysis showed that PtNi NPs were uniformly distributed in the PTOC nanocages with average particle sizes of 1.68 nm (Figure 3b). Further, high-resolution HRTEM analysis revealed that the d-spacing of 0.223 nm is between the (111) crystal faces of Pt (0.227 nm) and Ni (0.204 nm), indicating the formation of PtNi alloy NPs (Figure 3c) [43]. The HAADF-STEM image also confirmed the formation of uniformly distributed PtNi NPs (Figure 3d). EDX analysis showed that the as-prepared nanocomposites consisted of Ti, N, Pt, and Ni (Figure 3e–h). These results indicate that the 3D structure of PtNi NPs encapsulated by PTOC nanocages have been successfully prepared.

**Figure 4:**
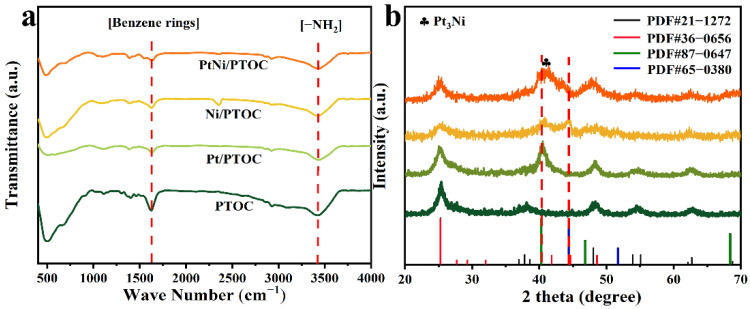
(**a**) FTIR spectra of catalysts. (**b**) XRD patterns of catalysts.

FT-IR measurements (Figure 4a) were carried out to detect the functional groups of the as-prepared catalysts. The experimental results showed that all the prepared catalysts contained benzene rings and amino groups (the characteristic peaks at 3428 cm^−1^ and 1630 cm^−1^). The existence of amino groups stably binds the metal NPs due to the strong chelation/complexation effect between the metal and amine groups [44]. Therefore, our results indicate that PTOC precursors are beneficial for the distribution of metal NPs. The XRD spectrum (Figure 4b) had four peaks at 2θ = 25°, 48°, 55°, and 62°, which corresponded to the (101), (200), (211), and (213) crystal planes of anatase TiO_2_ (PDF, No. 21-1272). The diffraction peaks of layered titanate H_2_Ti_8_O_17_ also appeared (PDF No. 36-0656), indicating that the PTOC had a two-component titanium oxide porous cage. In addition, the reflected signals of the Pt/PTOC and Ni/PTOC samples matched well with metallic Pt (PDF No. 87-0647) and Ni (PDF No. 65-0380), respectively. The diffraction peaks of PtNi alloy laid between the corresponding characteristic peaks of Pt and Ni, which further reflected the well-alloyed PtNi nanoparticles [45].

**Figure 5:**
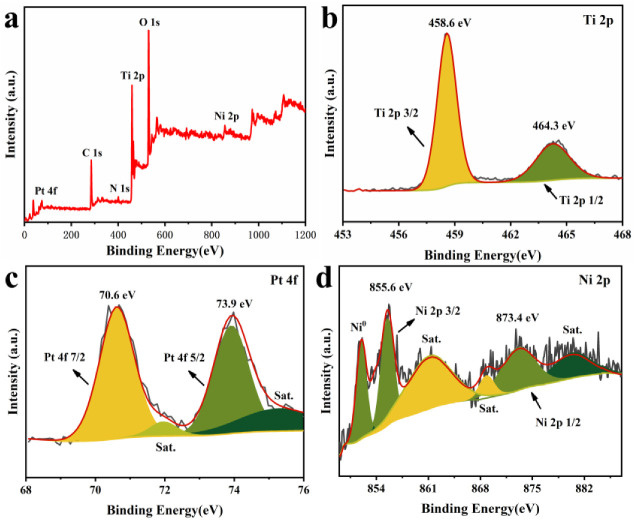
X-ray photoelectron spectra of Pt-Ni/PTOC catalyst (**a**) full spectrum; (**b**) Ti 2p; (**c**) Pt 4f; (**d**) Ni 2p.

The surface interactions and electronic state of PtNi/PTOC were investigated using XPS. Figure 5a shows the whole XPS pattern of PtNi/PTOC. The signals generated by the PTOC corresponded to C 1s, N 1s, O 1s, Ti 2p, Pt 4f, and Ni 2p. The narrow range spectra of Ti 2p is depicted in Figure 5b, which also proves the presence of PTOC. In Figure 5c, it can be seen that the Pt 4f region of core level binding energies is deconvoluted into two sets of spin-orbit doublet peaks. The Pt 4f spectrum exhibited two peaks at 71.4 and 74.7 eV and were assigned to Pt 4f7/2 and Pt 4f5/2, respectively, suggesting the presence of Pt^0^. Two peaks at 71.9 and 75.3 eV corresponded to the satellite peaks of Pt. The binding energy located at 855.6 and 873.4 eV belonged to the Ni 2p3/2, and Ni 2p1/2, respectively. The binding energies at 861.38, 868.92, and 873.4 eV corresponded to the satellite peaks. In addition, the binding energy around 852.05 eV is attributed to the Ni^0^ peak, which confirmed the existence of metallic Ni in PtNi/PTOC (Figure 5d). The strong interaction between Pt and Ni within the catalyst may lead to an increased oxidation resistance, which is beneficial to the catalysis activity and durability [46].

**Figure 6:**
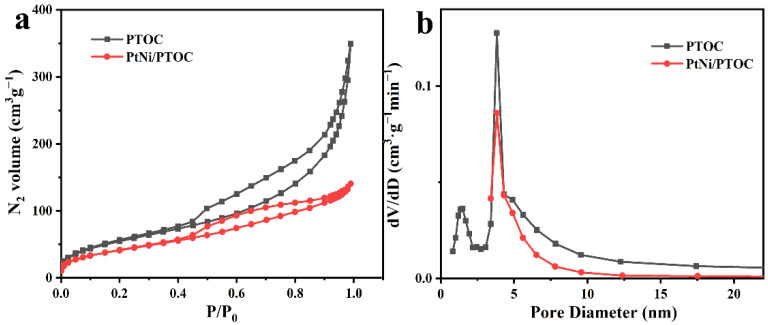
N_2_ desorption/adsorption isotherm (**a**) and pore-size distributions (**b**) for PTOC and PtNi/PTOC.

As shown in Figure 6a, PtNi/PTOC exhibited a typical IV-type isotherm with obvious hysteresis loops with a high specific surface area of approximately 206.2 m^2^·g^−1^. In addition, PTOC showed that similar isotherms with the specific surface area decreased from 206.2 m^2^·g^−1^ to 145.8 m^2^·g^−1^, which is due to the addition of PtNi NPs. From the IV-type isotherms with obvious hysteresis loops, the main pore size distribution of two materials is mesopores. Pore-size distribution curves showed that the size of the pore in PtNi/PTOC ranged from 3.5 nm to 10.0 nm (Figure 6b). The rich mesopores are conducive to the penetration of the electrolyte and the transport of electrons, thereby enhancing the catalytic activity of the material.

### 3.2. Effect of Different Types of Catalysts

**Figure 7:**
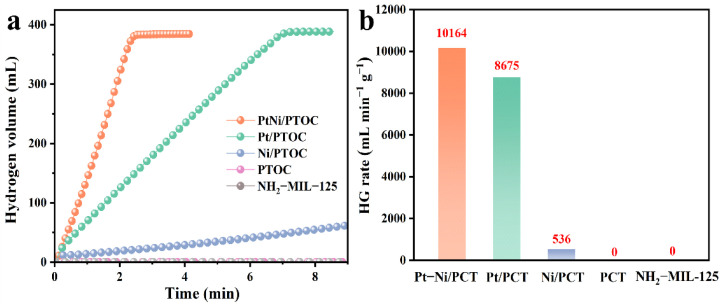
Hydrogen volume versus time (**a**) and HG rate bar chart of NH_2_-MIL-125, PTOC, Pt/PTOC, Ni/PTOC, and (**b**) PtNi/PTOC (reaction conditions: batch system, 25 °C, 1.5 wt% NaBH_4_ + 5 wt% NaOH, 0.1 g catalyst).

The effect of different catalysts on the hydrolysis of NaBH_4_ under alkaline conditions was investigated. As shown in Figure 7, PtNi/PTOC exhibited optimal performance with hydrogen release rate (HGR) of 10,164.3 mL·min**^−^**^1^ at 25 °C, which is higher than Pt/PTOC and Ni/PTOC. Compared to most of the previously reported results, PtNi/PTOC also exhibited a good catalytic activity (Table 1) [25,47,48,49,50,51,52]. According to Figure 7b, the magnitude of the catalytic performance was PtNi/PTOC > Pt/PTOC > Ni/PTOC, while PTOC and NH_2_-MIL-125 had no catalytic activity. The experimental results show that the synergistic effect between Pt and Ni enhanced the catalytic activity more than the single Pt or Ni-based catalyst, thereby promoting the rapid release of hydrogen from NaBH_4_. Furthermore, the evenly distributed PtNi alloy particles with more catalytically active sites simultaneously enhanced the hydrolysis activity.

**Figure 8:**
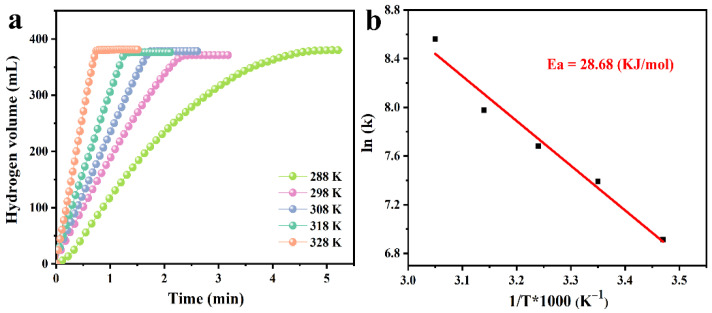
(**a**) Hydrogen generation kinetics curves and (**b**) Arrhenius plot obtained using 1.5 wt% NaBH_4_ and 1.0 wt% NaOH solution and employing PtNi/PTOC as a catalyst at different solution temperatures.

In order to measure the activation energy (*E_a_*) of the hydrolysis reaction, hydrolysis tests were carried out using different temperatures with the other parameters unchanged, controlling the reaction temperature from 15 to 55 °C (Figure 8a) with a gradient of 10 °C. As expected, all of the tests reached the theoretical hydrogen quantity, and the hydrogen release rate increased with the increase in reaction temperature, which belongs to the first-order reaction [53,54,55]. According to the Arrhenius slope calculation, the activation energy of Pt-Ni/PTOC is 28.7 kJ∙mol^−1^ (Figure 8b), which was lower than most of the catalysts that have been reported (Table 1). The synergistic effect between PtNi NPs and PTOC may be the main factor for the decrease in the *E_a_* value. The small particle size of the PtNi NPs is well supported on the pores of PTOC, thus avoiding excessive losses and agglomeration during hydrolysis. Moreover, the porous hollow structure promotes the interaction mass transfer between the catalyst and NaBH_4_ in the pores. Therefore, these results show that PtNi/PTOC has good kinetic properties for catalyzing NaBH_4_ hydrogen release.

### 3.3. Stability of PtNi/PTOC

**Figure 9:**
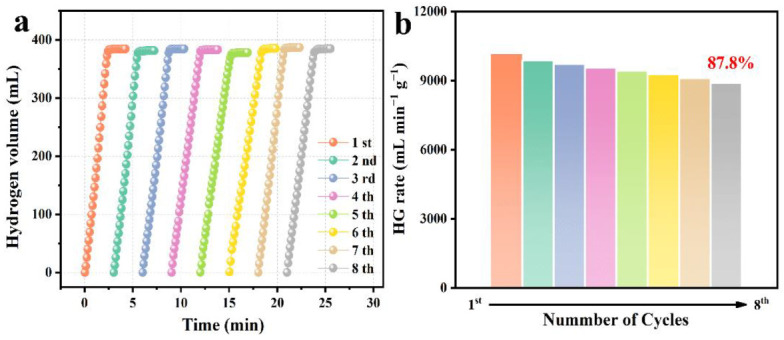
(**a**) Reusability of PtNi/PTOC with 0.1 g catalyst and 1.5 wt% NaBH_4_ + 5 wt% NaOH solution at 25 °C; (**b**) HG rate bar chart of catalyst used 8 times.

The stability of the catalyst is a key index to the actual application of hydrogen generation from NaBH_4_ hydrolysis. Figure 9 shows that the PtNi/PTOC catalyst was tested eight times under conventional conditions (25 °C). The catalytic activity of the hydrogen evolution of NaBH_4_ decreased slightly and maintained the initial catalytic activity of 87.8% after eight cycles. The excellent cycling performance may be related to the hollow porous structure of PTOC, not only providing a large surface area for the distribution of PtNi alloy particles but also suppressing the agglomeration issues.

**Figure 10:**
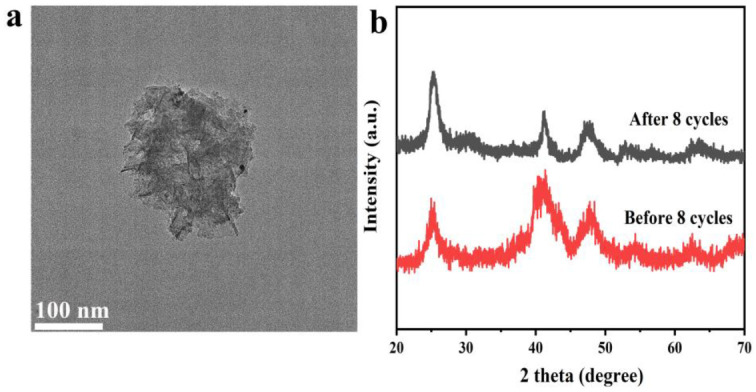
(**a**) TEM images of the PtNi/PTOC catalyst after 8 cycles; (**b**) XRD patterns of the PtNi/PTOC catalyst before cycling and after 8 cycles.

To verify the structural stability of the PtNi/PTOC catalyst, TEM (Figure 10a) and XRD characterizations were carried out after the cyclability test (Figure 10b). The TEM images of PtNi/PTOC after cycle tests show that the material maintained the nanocage structure with numerous sheets, indicating the stable structural integrity of the catalyst. In addition, compared to the original PtNi/PTOC sample, there was no significant agglomeration, which is favorable for the catalytic reaction. The XRD spectra of the obtained products showed that two XRD spectra were well-matched, and the peak of the catalyst became sharp after cycle tests, indicating the increased crystallinity of the catalyst. According to the Scherrer formula, (D = Kλ/βCosθ), the size of PtNi/PTOC catalyst increased from 1.68 to 2.32 nm after eight cycle tests, which was one of the reasons for the decay of catalytic activity. The stable structure and high catalytic activity of metal NPs are promising for the hydrolysis of borohydride.

## 4. Conclusions

In this work, ultra-small PtNi NPs were confined in a porous titanium oxide cage (PTOC) derived from NH_2_-MIL-125 (Ti) by a facile hydrothermal method and used for the hydrogen production of hydrolysis NaBH_4_. At a room temperature of 25 °C, the hydrogen production rate of PtNi/PTOC reached 10,164.3 mL·min^−1^·g_M_^−1^, and the activation energy was 28.7 kJ∙mol^−1^. After eight cycles of testing, 87.8% of the initial test performance was maintained. Such excellent performance can be attributed to the following: (i) The porous and hollow structure of PTOC creates a unique microenvironment between its interior and exterior, which provides more reaction channels. (ii) PTOC with a high surface area enables the even distribution of PtNi alloy particles, thus exhibiting a large number of active sites. (iii) The synergistic effect between PTOC and PtNi alloy particles can improve the reactivity. (iv) The robust porous structure maintains the integrity of the catalyst and suppresses the aggregation of nanoparticles. The catalyst has the advantages of a simple operation and economic efficiency and shows promise for producing hydrogen for fuel-cell vehicles.

## Figures and Tables

**Table 1:** Comparison of catalyst systems, reaction temperatures, HGR, *Ea* values, and number of cycles for NaBH_4_ hydrolysis catalyzed by various catalysts.

Sample	Tempera-ture (°C)	HG Rate (mL·min^−1^·g_M_^−1^)	*E_a_* (kJ·mol^–1^)	Number of Cycles	Cyclic Stability	Ref.
CNSs@Pt_0_._1_Co_0_._9_	30	8943.0	38.0	5	85.2%	[25]
Pt/MWCNTs	30	16.9	46.2	5	80.0%	[47]
Pt/CeO_2_-Co_7_Ni_2_O_x_	25	7834.8	47.4	5	85.0%	[48]
PtPd/GO	25	3940.0	29.4	4	60.0%	[49]
Pt/Si_3_N_4_	80	13,000.0	35.2	5	82.5%	[50]
NiCoP NA/Ti	30	3016.8	52.7	8	70.0%	[51]
RuNi/Ti_3_C_2×2_	30	1649.0	34.7	4	50%	[52]
PtNi/PTOC	29	10,164.3	28.7	8	87.8%	This work

## Data Availability

Not applicable.
